# Management of Fallot's Uncorrected Tetralogy in Adulthood: A Narrative Review

**DOI:** 10.7759/cureus.67063

**Published:** 2024-08-17

**Authors:** Anne Elizabeth Kaiser, Muhammad Ammar Husnain, Laraib Fakhare Alam, Siva Kumar Murugan, Rajanikant Kumar

**Affiliations:** 1 Research, Universidad Autónoma de Guadalajara School of Medicine, Guadalajara, MEX; 2 Internal Medicine, CMH Lahore Medical College and Institute of Dentistry, Lahore, PAK; 3 Department of Internal Medicine, Ministry of Health, Kuwait, KWT; 4 Department of Medicine, Meenakshi Medical College and Research Institute, Kanchipuram, IND; 5 Cardiothoracic Surgery, Medanta Superspeciality Hospital, Patna, IND

**Keywords:** pulmonary-valve replacement (pvr), heart failure, biventricular connection of the aortic root, ventricular septal defect, cyanotic congenital heart disease, tetralogy of fallot (tof)

## Abstract

The majority of cyanotic congenital cardiac defects are caused by the tetralogy of Fallot. Some symptoms include a biventricular connection of the aortic root, right ventricular hypertrophy, blockage of the right ventricular outflow tract, and a ventricular septal defect. Our understanding of tetralogy of Fallot (TOF) has significantly advanced since it was first described in 1888, and early diagnosis has led to improved surgical management and increased life expectancy. Adults with unrepaired and repaired TOF present with a range of late complications, including heart failure, the need for re-interventions, and late arrhythmias. Right ventricular (RV) failure, often caused by chronic pulmonary regurgitation, is a significant cause of heart failure in patients with TOF. Current treatment options are limited, and mainstay surgical procedures such as pulmonary-valve replacement (PVR), trans-annular repair (TAR), or infundibular widening repair have not shown a significant reduction in preventing right ventricular (RV) failure or death. Here, we explain the mechanisms of RV failure in ToF, chronic pulmonary regurgitation, heart failure, and secondary polycythemia. HF management in untreated adults is discussed. The progression of the disease, as well as complications, are also discussed. The treatment plan and the need to investigate the best management approach for this unsolved problem are included. This review aims to fill the knowledge gaps and supply valuable information regarding mechanisms of RV failure, chronic pulmonary regurgitation, and secondary polycythemia. To summarize, a new combat strategy must be found to battle RVF, and a more profound vision of these mechanisms is required. If it is not corrected, it will be one of the future research lines that will contribute to designing more efficacious treatment techniques for adults with TOF.

## Introduction and background

A cyanotic congenital heart disease, the tetralogy of Fallot, affects around one out of every three thousand live infants [1]. The Fallot tetralogy's physiological effects were dubbed "Louis Arthur Etienne Fallot's 1888 work "La maladie bleue" [2]. Right ventricular hypertrophy, blockage of the outflow path from the right ventricle, aortic root biventricular connection that crosses the muscular ventricular septum, and ventricular septal defect are the characteristic hallmarks [3]. Over the last 130 years, since Fallot described the hallmarks of "la maladie bleue," there has been significant progress in our knowledge of developmental abnormalities, phenotypic characteristics, and surgical treatment. The antero-cephalad deviation of the outflow septum and concomitant aberrant septo-parietal trabeculations are now widely recognized as diagnostics of tetralogy [4,5]. Aortic override of varied degrees is another consequence of the outlet septum's deviation, linked to various forms of ventricular septal defect [6].

Red cell mass (secondary polycythemia) and hemostasis are components of cyanotic congenital heart disease, which, like TOF, is a multisystem systemic condition [7-10]. As a protective mechanism against arterial hypoxemia, secondary polycythemia increases the red blood cell count. To induce erythropoiesis, the kidneys secrete erythropoietin. At increased hematocrit levels, a state of balance is achieved. As a defense mechanism against hypoxia, erythrocytosis increases red blood cell production. Iron-deficient erythrocytosis increases the risk of thrombosis of the cerebral venous sinuses in cyanotic children under four years old [7]. Paradoxical emboli may induce strokes and transient ischemic episodes via the right-to-left shunts and have congenital cardiac defects characterized by cyanosis.

Patients diagnosed with cyanotic congenital heart disease have been shown to have hemostatic problems. There are several clotting factor diseases, including anomalies of the von Willebrand factor that have recently been discovered, and platelet levels are low compared to normal [8]. Menorrhagia, bruising easily, epistaxis, bleeding from the gums, and an increased likelihood of traumatic bleeding are all symptoms of mucocutaneous bleeding. The most dangerous kind of bleeding, pulmonary hemorrhage, may be either external (hemoptysis) or intrapulmonary (intrapulmonary), and it can range from minor and infrequent to frequent, large, and deadly [9].

In the current era, there is a trend towards early surgical correction, which has significantly brought down mortality. At the same time, average life expectancy has also improved [10,11]. This has presented us with a growing cohort of survivors with repaired TOF, some of whom exhibit several late sequelae, viz., heart failure, the need for re-interventions, and late arrhythmias [12]. The most common cause of death in patients with the repaired tetralogy of Fallot (rTOF) is heart failure, which is the major area of focus in research [13,14]. In most instances, right ventricular failure is the underlying cause of heart failure in TOF. This is often the result of persistent pulmonary regurgitation, a typical consequence of treatments for pulmonary stenosis [13].

Currently, the medical treatment options for RV failure are lacking [14,15]. The most common surgical treatment option for patients with PR is pulmonary valve replacement. However, there is no clarity as to when valve replacement should be done, partly because repeat re-interventions may be needed because of child growth and prosthetic valvular degeneration [16]. A better knowledge of the transition from adaptive remodeling to failure is necessary to find new therapy targets for RV failure. The current understanding of RV failure mechanisms in chronic PR, TOF, and secondary polycythemia is the primary goal of this narrative review, as well as to fill the current knowledge gaps regarding their mechanisms and better management.

## Review

Anatomy

The tetralogy of Fallot is a cyanotic congenital malformation of the heart, and it is described as a constellation of four primary lesions, which are as follows: The right ventricle is made up of three structures: at the entrance, which includes the chordae tendineae, papillary muscles; and at the base of the heart, the densely trabeculated myocardium; and at the top, the pulmonary valve and either the infundibulum or the conus make up the outflow, which is the area that connects the RV cavity to the pulmonary trunk [17].

The trabeculations are nothing but muscle bundles that are arranged in a crisscross manner inside the cavity. They are seen in the apical 1/3 to 1/2 part of the RV. These muscle bundles in the right ventricle are coarser than those in the left ventricle. One of the essential trabeculations is the septo-marginal trabeculation, a Y-shaped muscle with a broad body and two divisions, or arms. The SMT lies adherent to the septum. One arm of the SMT travels one approaches the pulmonary valve from the front, and the other from the back, approaching the tricuspid valve. A muscular septum called the outlet septum is attached to the cephalad arm of the SMT. Separating the sub-aortic outflow tract from the sub-pulmonary infundibulum-another name for the sub-pulmonary outflow tract-is the outlet septum.

**Figure 1:**
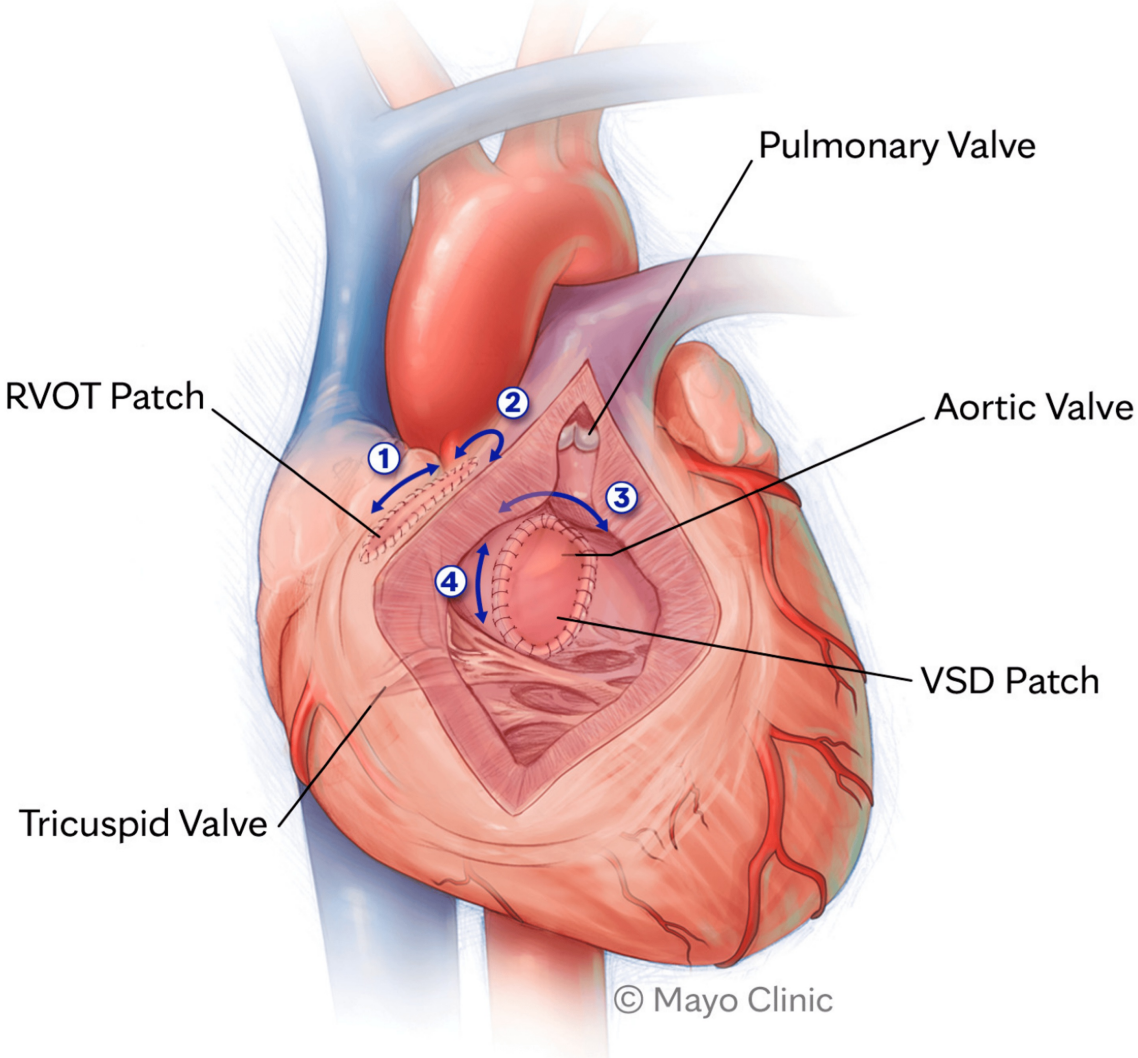
Ventricles are tachycardia-inducing anatomic isthmuses The acronyms "RVOT" and "VSD" stand for deficiency in the wall between the ventricles of the heart. Reproduced under the terms and conditions of the Creative Commons Attribution (CC BY) license from reference [19]. Copyright © 2023 by the authors. Licensee: MDPI, Basel, Switzerland.

The anterior and cephalad misalignment of this outlet septum is the hallmark feature of TOF. The misalignment of the outlet septum with varying degrees of hypertrophy of SMT causes the RVOTO [18]. Right ventricular outlet obstruction is present in the sub-infundibular region in around 50% of the cases. However, in some cases, the obstruction can also be at the level of the pulmonary valve or a mixture of the two [19]. Between the septo-marginal trabeculation's two arms is where the ventricular septal defect is located in TOF. The misalignment of the outlet septum axis with the ventricular septum axis causes the defect. The misaligned outlet septum, which causes the RVOTO, forms the roof of the defect. In most cases, the defect is peri-membranous; however, muscular defects of VSD are not uncommon in TOF. The ventricular septal defect is a non-restrictive defect, meaning the blood flow can occur in both directions across the VSD depending on the pressure (Figure 1) [19].

The RVOTO causes TOF right ventricular hypertrophy. An increase in intraventricular pressure causes myocardial hypertrophy to compensate for reduced blood flow from the right ventricle to the pulmonary artery as a result of the blockage. When the exit septum is not in the correct position, the aorta might pass over the VSD, a condition known as the overriding aorta [20]. The aorta in TOF still arises from the LV, although it can also receive blood from the RV. Due to the RVOTO and a non-restrictive VSD, a significant amount of deoxygenated blood goes to the aorta, which causes the aorta to be in a chronic volume overload state. This can be seen as a dilated aortic root in adults with TOF.

Epidemiology and incidence in adult life

Tetralogy of Fallot is seen in three out of 1,000 live births, which is equivalent to around 10% of all congenital heart defects. When we investigated the incidence of TOF in adults approximately 50 years ago, a study of TOF revealed that 17.6% of the participants were older than 25 [21]. However, with recent advancements in diagnosis, including prenatal and neonatal screening and advanced treatment options, the incidence of uncorrected TOF in adults has significantly decreased. It is now reported that 10% of the affected patients survive to adulthood, and 5% have a life span of 40 years or more [21].

Overview of heart failure and secondary polycythemia as complications of TOF

**Figure 2:**
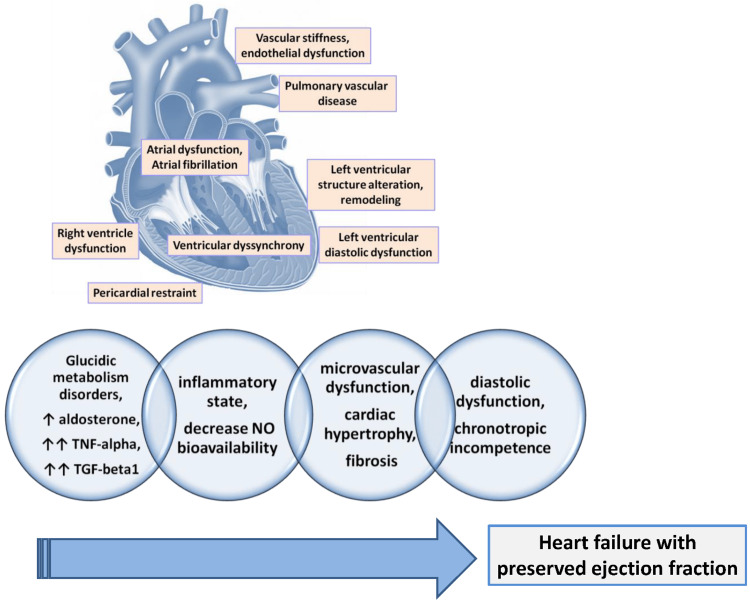
The key physiopathological pathways that lead to HFpEF Reproduced under the terms and conditions of the Creative Commons Attribution (CC BY) license from reference [23]. Copyright © 2024 by the authors. Licensee: MDPI, Basel, Switzerland.

The majority of individuals with TOF have right-sided heart failure. Pressure and volume overload are two potential causes of heart failure in TOF. In the post-natal period in an otherwise normal neonate, the right-sided pressures decrease after the separation of pulmonary and systemic circulation. However, in TOF, RV's pressure remains elevated due to RVOTO. This increase in pressure causes RVH and subsequently leads to diastolic dysfunction due to impaired filling, causing HFpEF. On the other hand, corrective surgeries that aim at alleviating the pulmonary stenosis can give rise to chronic pulmonary regurgitation, which can culminate in a systolic dysfunction of the RV, causing an HFrEF [22]. Secondary polycythemia in TOF occurs to compensate for the right-to-left shunt that causes hypoxemia. The resultant tissue hypoxemia increases the erythropoietin levels, which then stimulate the bone marrow precursors, causing increased RBC mass and hematocrit. This improves the oxygen-carrying capacity to some extent. However, the increased RBC mass also increases the blood viscosity level, which can cause decreased blood flow and tissue hypoxia. Increased blood viscosity levels can predispose the patient to a variety of thrombotic conditions and can also cause a state of iron deficiency anemia due to the rampant use of iron stores for erythropoiesis (Figure 2) [23].

Patients with secondary polycythemia can be divided into two groups: The first group includes people in a compensated phase of erythrocytosis who have sufficient iron reserves and few or no indications of hyperviscosity. The decompensated phase is part of the second set, with recurrent hyperviscosity symptoms and iron deficiency anemia [24].

Clinical manifestations of tetralogy of Fallot in adults

Tetralogy of Fallot is a congenital cyanotic heart condition that requires surgical repair or intervention as early as the first year of life. Though rare, this condition remains to survive to maturity without surgical intervention; it is the most prevalent congenital cyanotic cardiac condition. The incidence is equal for males and females [25]. It proceeds to progression and worsening of symptoms in adulthood when left untreated. Less than 5% of patients survive to 30 years and older without surgical intervention. The syndrome mainly characterizes the following features: First, a supremating Aorta Two, a defect in the ventricular septum Third, a blockage to the right ventricular outflow Fourth, an enlarged right ventricle [26].

In adults, the distinctive features include a significant ventricular septal defect, a greater incidence of combined pulmonary valve and infundibular stenosis, and congestive heart failure, which are seen in 33% of people with cyanosis compared to 38% in people without cyanosis. Whether cyanosis is present, early adulthood is also characterized by chest discomfort. In the second decade of life, there is a time when there are no symptoms. Pulmonary vascularity is either normal or increased in about 42% of the patients with cyanosis, whereas the absence of hypervascularity is seen in 70% of patients without cyanosis [27]. Right ventricle hypertrophy is the outcome of the large VSD and RVOT obstruction, leading to systemic right ventricle systolic pressure [28]. “Tet” spells are used for episodic hyper-cyanotic spells that start in early childhood with unrepaired TOF due to transient occlusion of the right ventricle outflow tract. The absolute cause of these spells is still unclear; however, some proposed mechanisms include increased hyperventilation, peripheral vasodilation, proper ventricle mechanoreceptor stimulation, and increased contractility of the infundibulum. As per a recent study, there is a high rate of cyanotic heart disorders in adults that may cause a stroke. Stroke occurred in 8.9% of males and 6.8% of women younger than 65 years old among about 30,000 adult patients with coronary heart disease. Ischemia stroke and hemorrhagic stroke occur at rates twelve and six times greater, respectively than in the general population, respectively, in adults with CHD. Ischemic strokes have been seen to perhaps be linked to diabetes, heart failure, and a recent myocardial infarction [29]. Abnormal lung function is commonly seen in adults with CHD, with symptoms [30,31]. Most liver symptoms are a result of hemodynamic consequences; for example, ischemic hepatitis or congestive hepatopathy are both caused by reduced blood flow to the liver [30-39]. Renal dysfunction is a common occurrence. A decline of 18-fold in GFR is seen compared to the whole population. There was mild renal impairment in 50.2% of the 1102 CHD patients analyzed in a cohort study, and moderate to severe decreased renal function in 9.3% [32]. There is evidence of an association of multiple syndromes with CHD. For example, Turner syndrome (with DM, estrogen deficiency, and hypothyroidism), DiGeorge’s syndrome, 22q11.2 deletion (with Grave’s disease and hypocalcemia), Downs syndrome (with DM and thyroid disorders), and Williams-Beuren syndrome (with hypothyroidism, DM, and hypercalcemia). There is an increased risk of thrombosis secondary to hematologic abnormalities and stasis (atrial arrhythmia, chamber enlargement). Patients with CHD have an increased risk of morbidity and mortality due to thrombosis and the shunting of blood seen in TOF [33].

Mortality in unrepaired tetralogy of Fallot

Individuals without surgical intervention are most often killed by hypoxia episodes (68%), cerebrovascular accidents (17%), and brain abscesses (13%). Without treatment, severe RVOTO kills 25% of infants in the first year, 40% by the third year, 70% by the 10th year, and 95% by the 40th year [34,35].

Before surgical repair became the norm, half of all Tetralogy of Fallot patients would die in their first few years, and even fewer would make it beyond their 30s. Surgical surgery has increased the current survival rate for individuals with isolated Tetralogy of Fallot from 8% to 92% beyond the age of 10. However, the degree to which pulmonary artery disease is severe does have a role. Prostaglandin medication helps maintain the duct open and prevents potentially deadly cyanosis in newborns with arterial atresia or severe stenosis [36,37].

In the third trimester of pregnancy, when pulmonary atresia or severe stenosis are present. The continuation of blood flow to the lungs is guaranteed by this flow. When ductal flow is retrograde, it means that you need to do something quickly. The goal of prostaglandin therapy after delivery is to keep the ductal arch open so that blood may flow backward into the pulmonary veins and oxygenate the lungs [38]. While some prestigious hospitals across the world delay heart surgery until the baby is around six months old, others begin the process immediately after delivery [39]. When a baby is six months old, the cardiologists at Melbourne's Royal Children's Hospital would rather wait to do a complete repair. Temporary shunt repairs may be necessary to get patients with severe cyanosis or hypoxia to a stage where a thorough repair may be done. In a pair closing the ventricular septal defect using a transannular patch fabricated from synthetic, bovine, or autologous material is the procedure of full repair. Also included is the possibility of using a second patch to enlarge the right ventricle and pulmonary artery's narrowed area [40]. Patients diagnosed with Tetralogy of Fallot have excellent long-term outcomes because of surgical procedure developments in the previous 50 years [41]. Although these operations increase the risk of sudden, unexplained cardiac arrest and left- and right-sided heart failure, they are nevertheless worth doing for a total of 29 Research done in 2019 found that the current survival rate for people above the age of 10 is over 92%, despite the danger of early death. Considering that the mortality rate was 50% in 1955, this is a notable improvement [36,37].

**Figure 3:**
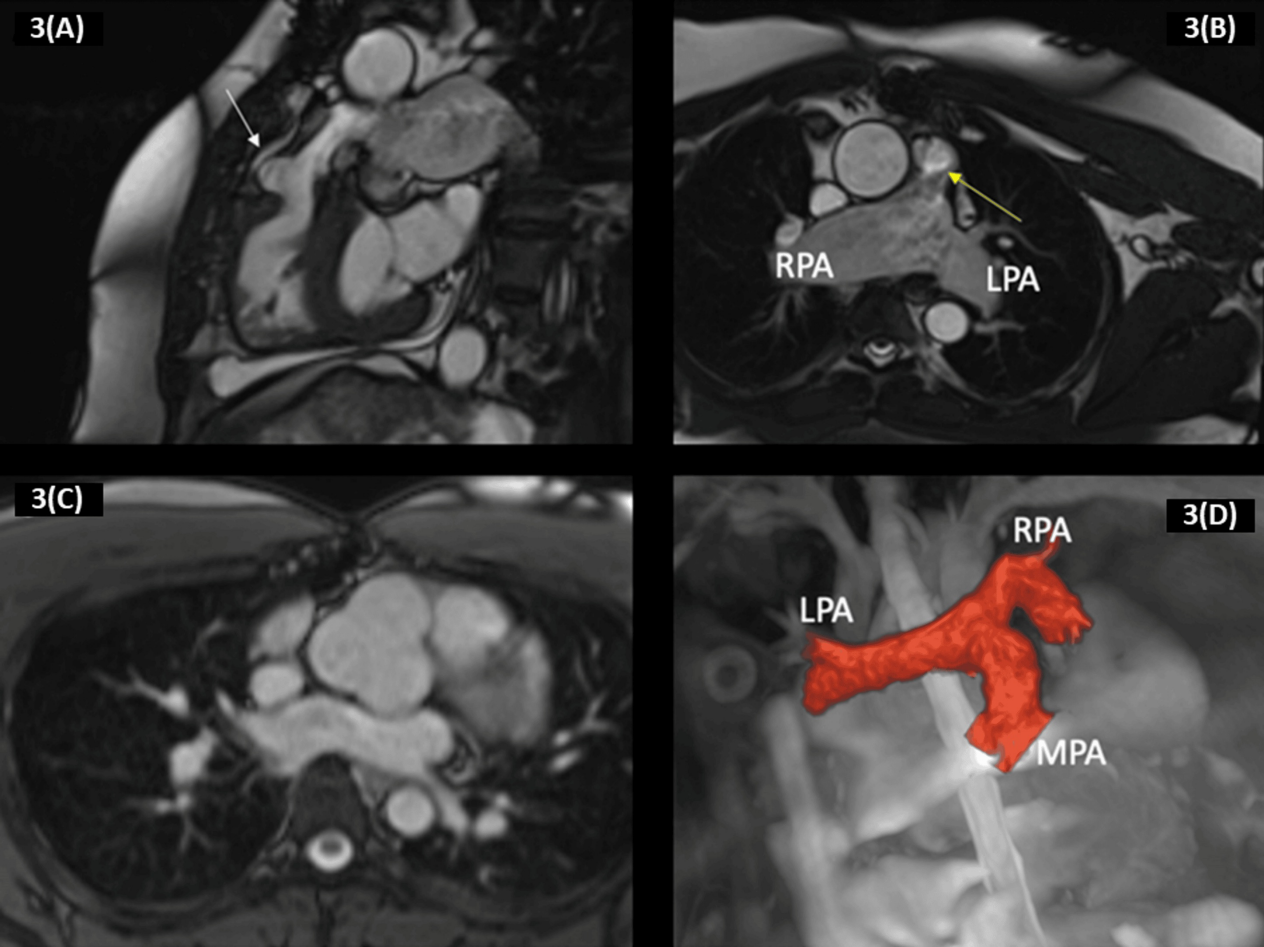
It depicts a 40-year-old patient who was born with the tetralogy of Fallot with a missing pulmonary valve. 3(A) The patient had total TOF surgery at the age of seven. At the age of 32, the patient had a pulmonary valve replacement using a 23-mm aortic homograft to address severe pulmonary regurgitation, as indicated by a white arrow. 3(B) A balanced steady-state free precession sequence provides a clear view of the right ventricular outflow tract with the patch employed during the repair, indicated by a yellow arrow. 3(C) A transaxial plane bSSFP cine sequence was obtained, showing dilated right pulmonary artery and left pulmonary artery, as well as modest accelerated flow in the primary pulmonary artery. The balanced steady-state free precession cine sequence demonstrates an enlarged aortic root. 3(D) Another patient was born with a tetralogy of fallot and had complete surgical correction during early childhood. The 3D complete heart sequence displays the architecture of the pulmonary arteries Reproduced under the terms and conditions of the Creative Commons Attribution (CC BY) license from reference [44]. Copyright © 2023 by the authors. Licensee: MDPI, Basel, Switzerland.

Cyanosis, if present, is visible at two significant sites: nail beds and lips. On physical examination, an RV impulse and a systolic thrill can be appreciated during palpation. Hepatomegaly is relatively uncommon. On most occasions, the peripheral pulses are regular. If prominent pulses are felt, they could suggest aorto-pulmonary collaterals or a prominent patent ductus arteriosus. Although TOF manifests primarily with cyanosis in childhood, it is seen in adults with exercise intolerance [42]. The majority of the patients left untreated died during early childhood. The leading causes of death are pulmonary embolism, brain abscess, and thromboembolism. Other manifestations can be seen, such as stroke, syncope, and seizures, with cerebral venous thrombosis being the most common. Unlike children, the risk factors for stroke in adults are hypertension, phlebotomy, diabetes mellitus, microcytosis, and rhythm disorders. Data suggests that, unfortunately, only 1% of the untreated patients survive up to the age of 50 years, compared to the increased survival rate of 74% in individuals who received surgical intervention early on in life [43]. As per the literature, the oldest patient with an uncorrected TOF was 86 years old and from the USA. Clinical manifestations, progression of symptoms, and prognosis in adults with TOF who didn’t receive surgical intervention are predominantly determined by infundibular stenosis, which narrows with age, and collateral circulation to the lungs, which develops in childhood. Both of these factors, combined, lead to an altered expression of TOF in adult life (Figure 3) [44].

Complications

Short-Term Complications

The recurrent laryngeal and phrenic nerves are at risk of harm when a modified Blalock-Taussig shunt is implanted during palliative surgery. Inadequate blood supply to the lungs, which may cause congestive heart failure (CHF), or too much blood flow, which can cause failure to thrive (cyanosis), can be caused by the size of the shunt [45]. Extracorporeal membrane oxygenation is a final option for patients with shunt thrombosis who have not yet had urgent reoperation or interventional catheterization for direct thrombolysis [46].

After tetralogy of Fallot surgery, there are a few potential complications that can arise. These include fluid buildup around the heart and lungs, which may need drainage; lymphatic fluid leaking into the chest cavity (chylothorax); additional surgery to control bleeding; superficial infection of the surgical wound; moderate backward blood flow in the pulmonary artery in approximately 14% of patients; persistence of ventricular septal defects causing low oxygen levels; and blockage of blood flow from the right ventricle [47].

Reduced cardiac output, severe hypotension, and unstable hemodynamics might occur as a consequence of junctional ectopic tachycardia (JET), which can occur in around 15% to 20% of patients soon after surgery. Inducing hypothermia to cool the patient, reducing the use of vasoactive drugs like dopamine, restoring atrioventricular synchrony by pacing the atrial rate over the ventricular rate, using magnesium sulfate, prescribing antiarrhythmic medications like amiodarone, procainamide, or esmolol, and administering sedation to manage pain are all potential treatment options. Extracorporeal membrane oxygenation may be necessary to treat patients with arrhythmias and restore their heart's capacity to pump blood if therapy fails [48]. Using inotropes, such as dopamine, following surgery and being less than six months old are significant risk factors for postoperative JET [49].

In the early days following surgery, patients who have a transarterial-transpulmonary approach are less likely to develop tachyarrhythmias and almost never need a permanent pacemaker to correct abnormal cardiac conduction, in contrast to those who undergo a right ventriculotomy. An atrioventricular block occurs in 3-5% of patients, bifascicular block in 8-12% of patients, and a right bundle branch block occurs at a high frequency as a consequence of conduction system injury. One typical postoperative consequence is right ventricle noncompliance. The expansion of the RV and its decreased relaxation capacity, together with the conduction system's damage-induced disruption of the coordination of the RV contractions, led to its occurrence. Furthermore, the transannular patch is created by removing contractile muscle fibers, which leaves the RV-free wall with a non-contractile patch. Avoiding beta-agonists, using beta-blockers, and keeping an eye on fluid balance may be necessary for the treatment of right ventricular dysfunction in the immediate postoperative period [45].

Some arrhythmias, including ventricular tachycardia, atrial fibrillation, flutter, and intra-atrial reentrant tachycardia, may occur after a patient has had their Tetralogy of Fallot fixated. Having a QRS duration greater than 180 milliseconds, being male, being older at the time of repair, and suffering a temporary complete heart block after the third day following surgery are some risk factors for ventricular arrhythmias and sudden cardiac death [50]. There is an increased risk of mortality after surgery in patients who were born prematurely [46]. Postoperative moderate-to-severe pulmonary regurgitation has been less common as a result of valve-sparing surgeries [47].

Pregnancy Complications

Results for women who had a tetralogy of Fallot repaired should be similar to those for the general obstetric population. Increases in pulmonary hypertension, pulmonary regurgitation, and right and left ventricular dysfunction are independent predictors of pregnancy complications. Atrial flutter, fibrillation, ventricular tachycardia, cardiac failure during the third trimester, and pulmonary embolism are all examples of arrhythmias that may occur during pregnancy and need medical attention. There have been several reports of complications in the offspring, including smaller than expected gestational age, premature birth, fetal death in the uterus, and an increased risk of congenital heart defects like tetralogy of Fallot, atrioventricular septal defect, and atrial septal defect [51].

Having a palliative shunt or moderate right ventricular hypertension increases the risk of cardiac events and miscarriage in pregnant patients. Unrepaired Tetralogy of Fallot (TOF) increases the risk of right ventricular hypertrophy, congestive heart failure, atrial and ventricular arrhythmias, and other complications in pregnant women. Postpartum hemorrhage and aortic root enlargement are two more possible consequences. Pulmonary hemorrhage, thrombosis, and brain abscesses are among the maternal causes of death that might affect this particular patient population. Instances of preterm delivery, low birth weight, and an increased risk of miscarriage are among the complications that may arise during pregnancy [52].

Long-Term Complications

Adults are experiencing a higher rate of CHD prevalence growth than children, with an estimated rate of around 5% per year. Therefore, it is critical to be aware of the possible complications that may develop after congenital heart defect surgery. It is well-recognized that pulmonary regurgitation may still worsen over time, even after valve-sparing surgeries. When the patient is 35 years old, after TOF surgery, the cumulative incidence of this syndrome exceeds 40%. The main cause of reoperation is pulmonary insufficiency. Impaired right and left ventricular performance and an increase in RV volume owing to pulmonary insufficiency are long-term consequences. Surgery or a transcatheter technique may replace a pulmonary valve [53].

Aortic root dilatation and insufficiency, ventricular hypertrophy, right ventricular aneurysm formation after ventriculotomy or the outflow patch, and distal pulmonary artery occlusion are additional long-term complications. When patients have exercise intolerance in addition to symptoms of congestive heart failure, the condition might be made worse by ventricular dyssynchrony [54]. When aortic insufficiency is present and the aortic root diameter is more than 55 cm, aortic root surgery is required. Endocarditis may occur in anybody with a Tetralogy of Fallot diagnosis, regardless of whether they have had surgical treatment or not. Subacute bacterial endocarditis is a serious health risk; hence, patients should take preventative measures against it before undergoing elective dental or surgical procedures.

Delays in the onset of aberrant cardiac rhythms (ventricular and atrial), heart failure, and complications from following surgical procedures are the main causes of mortality in patients who have had surgical correction for Tetralogy of Fallot. Ventricular arrhythmias, such as prolonged ventricular tachycardia with syncope, may occur in around 40 to 50% of patients who have had surgical correction for tetralogy of Fallot. Unexpected death rates range from 6% to 9% in the 30 years after surgery. Hypertension, a history of syncope, multifocal premature ventricular contractions, ventricular tachycardia, an advanced age (greater than 3 years), significant regurgitation of the pulmonary or tricuspid valves, and a QRS duration longer than 180 milliseconds are all associated with an increased risk of this condition [55].

As people with TOF get older, the risk of atrial flutter and fibrillation rises dramatically, which may cause serious health complications. Mild tricuspid regurgitation and a dilated right atrium are associated with atrial tachyarrhythmias. There is some evidence that atrial tachyarrhythmias and sinus node dysfunction may coexist and even maintain each other [56]. Additionally, adults with right ventricular to pulmonary artery conduits have an increased risk of death or prolonged ventricular tachycardia. Patients with coronary heart disease are at increased risk for complications and mortality due to metabolic syndrome, high blood pressure, obesity, and type 2 diabetes [57].

Neurodevelopmental dysfunction is a known risk for patients with congenital heart disease for a variety of reasons, including underlying syndromes, genetic or developmental issues, and medical and surgical interventions. These people often have mild cognitive impairment, problems with social interaction and communication, an inability to focus, impulsivity, and poor decision-making and planning skills. Physiotherapy, occupational therapy, speech therapy, and special education services are often required by many people. Many of these individuals have ongoing needs for these services well into adulthood. Their ability to receive health insurance, advance in their careers, and enjoy a high quality of life might all be hindered by these issues [58].

Diagnostic evaluation for tetralogy of Fallot in an adult patient

Standard Laboratory Evaluation

The recommended tests to include in a comprehensive blood test are a complete blood cell count, an iron study, and an assessment of vitamin levels such as vitamin B12 and folic acid. If the mean corpuscular volume is normal or elevated and there is evidence of iron deficiency. As part of this routine, checking creatinine levels, electrolytes, uric acid, and biomarkers such as BNP and NT-proBNP is essential [59].

Routine blood tests rule out iron deficiency anemia and evaluate prognostic outcomes. The drop in oxygen saturation and supply to the tissue kicks off a sequence of adaptive processes [60]. The kidneys release erythropoietin, which stimulates the production of red blood cells in the bone marrow. This is a natural response to low oxygen levels in the body. The amount of red blood cells/hemoglobin is inversely related to the oxygen content: as the oxygen saturation decreases, the hemoglobin levels increase.

Anemia caused by iron deficiency is often overlooked or not identified in patients with cyanosis because a hemoglobin level of 14 g/dL, considered normal for people without cyanosis, is misunderstood as usual for patients with cyanosis. We need a hemoglobin concentration of 20 g/dL or greater since the patient's oxygen saturation of 85% suggests severe anemia [61].

An exact description of the particular rise in hemoglobin and red blood cells is secondary erythrocytosis. The specific increase of red blood cells is often referred to as polycythemia. Be that as it may, this term is reserved for a clonal stem cell condition affecting platelets, white blood cells, and red blood cells. In contrast, patients with cyanotic congenital heart disease typically have an average white blood cell count and a lower platelet count. Therefore, it is recommended to avoid using the term polycythemia in patients with CCHD.

Coagulation profile accuracy may be compromised when analyzing a patient's blood with a hematocrit level over 55%. The patient's arterial blood gas analysis, which includes measurements of base excess, oxygen partial pressure, and serum lactate, indicates that their serum lactate levels, base excess value, and oxygenation status may serve as prognostic indicators for death in patients undergoing surgical correction of the tetralogy of Fallot [62].

Blood Biomarkers

Blood biomarkers have emerged as a promising tool for assessing the risk of complications in patients with congenital heart disease. Blood biomarkers and several pathways associated with remodeling, vascularization, and myocardial fibrosis have been identified recently, mostly in those who have had cardiac problems as adults [53,54]. However, only a limited number of tetralogies of Fallot individuals in their twenties have been the subjects of biomarker studies [63]. NT-proBNP is released in response to increasing ventricular volume, pressure overload, and myocardial stress. It is a popular biomarker for adult patients suffering from congestive heart failure or acquired heart failure; increased levels of NT-proBNP are related to mortality and adverse outcomes. A study conducted by researchers confirmed the above finding that elevated levels of NT-proBNP in patients with TOF were linked to the severity of pulmonary regurgitation and adverse events [64].

A family of proteins known as insulin-like growth factor-binding proteins regulates IGF activity and, by extension, growth hormone. Endothelial cells have a high expression level of IGFBP-7, which has been associated with the process of collagen deposition. IGFBP-7 has been related to the process of repairing the myocardium after a heart attack. IGFBP-7 has been recognized as a possible biomarker for worse outcomes in individuals with acquired heart failure. It is linked to diastolic dysfunction and a reduced VO2 max [65]. The impact of IGFPBs on cardiac function or prognosis in ConHD patients remains largely uninvestigated. However, it has been associated with failure to thrive, subclinical renal and nutritional status, and overall growth damage. In the same way, a greater ratio of RV mass to volume and worse outcomes were linked to elevated IGFBP-7 levels, according to a study conducted by Van Den Bosch E et al. [63]. Based on these findings, IGFBP-7 may play a role in the follow-up of patients with the tetralogy of Fallot and potentially other forms of congenital heart disease.

Fatty acid-binding protein-4 has a significant expression in adipocytes, and increased concentrations of FABP-4 are linked to diabetes, hypertension, obesity, and female gender. Macrophages display FABP-4 expression, which is believed to enhance the production of foam cells and trigger an inflammatory reaction. Hypertrophy of the left ventricle and dysfunction of the systolic and diastolic processes have been linked to FABP-4 levels. Adverse outcomes during follow-up were also associated with greater FABP4 levels in individuals with chronic heart failure. Compared to individuals with a VSD, in RV tissue, patients with TOF have elevated levels of FABP-4 RNA expression. The study conducted by Van den Bosch E et al. in the TOF cohort found a significant inverse correlation between FABP-4 levels and peak VO2. As such, we could assume that there is still more research to be done in order to shed light on the role of FABP4 concentrations in ConHD [66].

Electrocardiography

**Figure 4:**
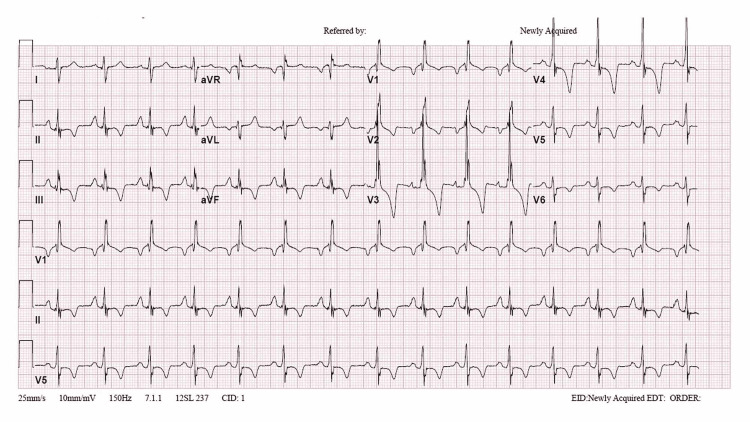
The patient's ECG shows a right bundle branch block and a strain pattern indicative of right ventricular hypertrophy Reproduced under the terms of the Creative Commons Attribution License CC-BY 4.0 from ref. [21]. Copyright© 2020 Alkashkari et al.

Right ventricular hypertrophy, accompanied by a right bundle branch block, is the most prevalent anomaly in electrocardiograms. A longer QRS interval would have increased the RV's volume and mass. Moreover, a QRS interval greater than 180 ms greatly increases the risk of ventricular tachycardia and abrupt cardiac arrest [67]. A researcher reported a case of an adult patient who was diagnosed with Tetralogy of Fallot (ToF). The lawsuit argues that individuals with primary congenital disorders may be untreated until they reach a more advanced age, despite advancements in medical knowledge and technology. Procrastinating the identification and treatment of ToF patients heightens the likelihood of unfavorable consequences. Nevertheless, our patient exhibited a remarkable post-operative and two-year follow-up profile. Conducting a comprehensive physical examination of neonates and performing an early-life screening echocardiogram may help in identifying the condition at an earlier stage (Figure 4) [21].

Imaging Studies

Echocardiography: The main imaging technique is transthoracic echocardiography, which is used in diagnosing and evaluating the tetralogy of Fallot both before and after surgery [68]. This modality is used at every stage of a patient's life, from diagnosing conditions before birth to assessing complications in older individuals. TTE is a cost-effective option that is widely accessible and has no reported detrimental effects on the body. The initial clinical management is typically guided by TTE, which may suffice as the sole imaging technique for follow-up in many patients. The importance of performing annual TTE in patients with significant PR or aortic root dilatation cannot be overstated. However, a less frequent follow-up every two years may be considered for asymptomatic patients with an uncomplicated progression.

Magnetic resonance imaging (MRI): The best way to assess the right ventricle's size and function is by magnetic resonance imaging and accurately measuring the volume of pulmonary regurgitation. An MRI is typically the initial test performed when there is suspicion of a problem with the pulmonary valve. The imaging modality visualizes the size of the aorta, the RVOT status, the pulmonary arteries, and the existence of VSDs and/or RV hypertrophy, and it maps the velocity of pulmonary regurgitation efficiently. Intracardiac pressures, gradients, and blood flows may all be measured using MRI.

Aortopulmonary collaterals and growing pulmonary valve insufficiency may be related to branch pulmonary artery stenosis, which MRI detects well. People who have both pulmonary atresia and the tetralogy of Fallot are more likely to have these outcomes [69].

CMR becomes more significant during adolescence. Baseline CMR should be performed on stable, asymptomatic TOF patients during the transition from pediatric to ACHD programs; after that, it should be routinely indicated every three years. Patients with advanced and complicated diseases are recommended to undergo CMR follow-up intervals every one to two years [70].

Cardiac CT: The primary benefit of CT is that multidetector CT offers the most superior spatial resolution among all imaging methods [71]. This enables a more comprehensive evaluation of the anatomy of small blood vessels, such as the pulmonary veins, peripheral branches of the pulmonary artery, and the collaterals of the aortopulmonary. Additionally, it is beneficial for imaging the coronary artery since 8-12% of individuals with tetralogy of Fallot have an abnormal coronary artery origin. CT is the preferred imaging modality for obtaining anatomical information in individuals who cannot undergo cardiac MRI due to contraindications [72].

Cardiac catheterization: Angiography has been the imaging gold standard for the preoperative evaluation of TOF for decades. Presently, developments in cross-sectional imaging enable the detection of anatomical details of extracardiac and intracardiac structures, such as the pattern of the coronary artery or the morphology of the pulmonary branches, in a non-invasive manner. Angiography by X-rays continues to be a viable alternative imaging modality for percutaneous interventions [73].

Pharmacological interventions and lifestyle modifications

When considering possible interventions for heart failure in adults with congenital heart disease, pharmacological and lifestyle modification-based approaches emerge as options. However, the tetralogy of Fallot would not benefit from regular medication use unless significant residual hemodynamic abnormalities were present [74].

Burchill and Webb further corroborated this in their 2015 study, in which they suggested that traditional neurohormonal medications such as angiotensin-converting enzyme inhibitors were ineffective in this setting as they failed to treat the physiological complications associated with the tetralogy of Fallot, including pulmonary regurgitation and right ventricular volume overload [75].

Instead, a more practical and reasonable approach may be to address modifiable lifestyle habits, such as tobacco and illicit drug use and diet, that impact the associated risks of developing obesity, diabetes, and hypertension. The merits of this strategy are primarily rooted in the concept of ventriculoarterial coupling. In other words, we must consider the combinatory role of increased arterial pressure and ventricular hypertrophy in mediating the development of diastolic heart failure. It naturally follows that making lifestyle changes that are proven to normalize arterial pressure may also prove beneficial in this scenario. Thus, lifestyle modifications such as a healthy diet and exercise may help regulate parameters like aortic diameter and stiffness, reducing the risk of secondary disease [76].

In addition to the conditions above, diseases that occur commonly in adulthood, such as acquired valvular heart disease, coronary artery disease, endocarditis, chronic respiratory disease, renal disease, obstructive sleep apnea, and hyperthyroidism or hypothyroidism, may act as triggering factors for heart failure in ACHD patients. Even factors such as pregnancy, cardiotoxic chemotherapy, and irradiation may contribute to increased risk. Given that ventricular dysfunction is often used as an accurate predictor of negative outcomes, frequent monitoring may effectively prevent adverse complications. Two such subclinical options include speckle tracking via echocardiography and myocardial deformation analysis via MRI [75].

Surgical interventions: indications, types, and outcomes

Surgical interventions form an essential part of the management of heart failure in the tetralogy of Fallot, especially when the patient is symptomatic with heart failure, cyanosis, or has different types of underlying arrhythmias. However, certain patients, including but not limited to those with pulmonary regurgitation, may be surgically intervened before symptoms arise, and even asymptomatic patients in whom medical efforts have failed give way to surgical intervention as the next treatment option [77].

In addition, the degree of right ventricular dysfunction, the severity of pulmonary insufficiency, and residual outflow tract obstruction in case of any previous repair also become deciding factors when taking into consideration surgical intervention for TOF-related heart failure.

Although various surgical interventions are available for the management of heart failure, the most commonly used is pulmonary valve replacement since it results in a huge enhancement in exercise capacity, quality of life, and heart failure symptoms. Moreover, it can also be performed in several ways, including minimal access via a small incision, percutaneous balloon pulmonary valvotomy, open-heart surgery, or stent implantation. Though valve replacement effectively reverses ventricular function, irreversible diastolic dysfunction sometimes persists. Second, trans-annular patch repair is another intervention undertaken in patients who already have a history of previous repair that led to residual outflow tract obstruction, which in turn caused increased pulmonary artery pressure, paving the way for RV dysfunction and heart failure. It aims to relieve the obstruction and works by enhancing RV function, leading to an excellent long-term outcome and alleviation of heart failure symptoms [78].

Despite the spectrum of treatment options available, including non-pharmacological lifestyle modifications like exercise, certain aspects must also be taken into account to weigh the risks and benefits of each treatment modality. For example, patients on an exercise regimen for heart failure in TOF must be monitored to avoid any overexertion and the potential for any complications that may lead to grave consequences. Another critical point to consider is the timing of intervention since, as age progresses, the patient becomes weaker, and the heart's anatomy can make even simple procedures difficult. Mechanical circulatory support should also be considered when all treatment efforts have failed to improve the RV function. At the same time, it becomes difficult to provide the MCS even in adulthood compared to childhood. Finally, risk stratification using different cardiovascular risk predictors such as right and left ventricular ejection fraction, right and left ventricular volume, preoperative peak oxygen consumption, right atrium systolic indexed volume, RV global longitudinal strain, and apical rotation must be accounted for to generate a treatment plan that optimizes the modality and leads to a better lifelong prognosis [79].

Management of secondary polycythemia

Polycythemia can result from myeloproliferative disorders, known as polycythemia-vera, or can be secondary to increased red blood cells regulated by erythropoietin. This kind is known as secondary polycythemia. SP is further categorized into two main types: acquired and congenital. The acquired form is further subdivided into erythropoietin-associated (hypoxia, pathological erythropoietin production) and non-erythropoietin-associated (post-transplant, drugs, etc.).

In TOF, we see a right-to-left shunt, which causes the mixing of oxygenated blood with deoxygenated blood, leading to acquired secondary polycythemia [SP] erythropoietin-associated hypoxia. Elevated erythropoietin and hemoglobin levels are identified as erythropoietin-mediated and hypoxia-dependent SP. However, SP is not dependent on hypoxia and has normalized erythropoietin levels after elevated hemoglobin levels [80]. Polycythemia is a blood condition with elevated hemoglobin and hematocrit.

In SP, when compared to PV, the rate and severity of thrombotic and non-thrombotic complications are relatively lower. This review will focus on three main categories for treating SP: phlebotomy, hydration, and medications. Each will be individually discussed below.

Phlebotomy

For SP patients with symptoms secondary to hyperviscosity, prophylactic phlebotomy is advised at moderate-severe symptoms and with a target hematocrit level (> 65) higher than the target level seen in PV. As per a study, an adult patient with uncorrected TOF and SP underwent regular phlebotomy sessions despite having an Hct level of <65%. A systematic review assessing the effectiveness of phlebotomy in SP demonstrated a clinical improvement in the symptoms. The majority of the patients either reported complete resolution or subjective improvement in their clinical state. The main target is to keep the hematocrit <55%. Even though it is rarely used, phlebotomy is still considered an effective and safe option for the symptomatic treatment of SP. However, prospective studies are required to determine its implications for objective improvement.

Dehydration

Dehydration can precipitate thrombosis in patients with SP secondary to CHD; therefore, the aim should be to keep patients well-hydrated precisely when they develop inter-current gastrointestinal disorders. Another precaution to be taken is to avoid prolonged fasting without adequate rehydration, as this can lead to dangerously high levels of hematocrit in patients who are already predisposed to hypercoagulability. Volume depletion with subsequent vasoconstriction can cause multi-organ ischemia and even intraoperative thrombus formation.

Medications

Another option is the use of cytotoxic drugs. Others, such as theophylline and enalapril, have also been used to reduce the hematocrit, mainly secondary to real transplantation as a result of renal abnormalities seen in CHD and theophylline alone in patients with SP with chronic obstructive lung disease as a complication.

Monitoring and management of complications

Dealing with SP in relation to CHD, we can encounter certain complications. The challenge is to identify, monitor, and manage them accordingly. The main complications we will address in our article are thrombosis, bleeding, and iron deficiency anemia.

Thrombosis

Patients with polycythemia are prone to thrombosis, mainly pulmonary or cerebral. Therefore, careful attention is needed in any preoperative evaluation of a patient's baseline blood profile, followed by tailored treatment strategies to prevent morbidity and mortality [80]. Cerebral venous thrombosis can be a serious neurologic condition, but it is potentially reversible with an immediate diagnosis and appropriate medical care. Imaging plays a major role in the diagnosis. MRI, CT, MR venography and CT venography are particularly useful [81].

Iron Deficiency Anemia

Due to repeated phlebotomies in SP patients, a rapid fall in the hemoglobin concentration was observed. There is an eventual, gradual fall in the plasma iron concentration. Subsequently, there is an increase in the total iron binding capacity. These changes persisted until the induced anemia; thus, Hb levels were returned to their pre-phlebotomy values. The following diagram shows the relationship between repeated phlebotomies and subsequent falls in hemoglobin levels [82].

Hemorrhage

Cerebral ischemia secondary to thromboembolic events and eventual hemorrhages, even though uncommon, are reported to be the main complications of SP. A case report was examined involving a 35-year-old male patient with SP who had subarachnoid hemorrhage as a result of cerebral vein thrombosis. On the second day of his neurological complaints, the patient had his first non-contrast CT, which revealed cerebral edema free of hemorrhage.

On day four, the patient underwent a second non-contrast CT scan. The results showed subarachnoid hemorrhage in the right parietal region (green arrows) and intracerebral hemorrhage in the right basal ganglia (yellow arrows), creating a midline shift to the left. Lastly, there was cerebral infarction in the right fronto-temporo-parietal region (blue arrows) with an ASPECT score of 1 [83]. Therefore, diagnostic imaging plays a significant role in the monitoring and management of patients with diseases with increased coagulation, such as SP, as in our discussion.

Tailored management for ToF patients requires specialists to analyze the patient’s risk of atrial fibrillation and ventricular tachycardia. A study reviewed atrial fibrillation in association with heart failure hospitalization in adult patients with corrected TOF. The study concluded that rhythm control therapy decreases heart failure hospitalizations and mortality in TOF patients. They predicted an 82% success rate of patients returning to normal sinus rhythm after direct DC cardioversion. Using the Vaughn-Williams categorization criteria, researchers determined whether patients in the rhythm control group had dosages of Class I and Class III antiarrhythmic medicines, switched to a different Class I or Class III AAD, or had catheter ablation surgery.

The follow-up consultations between the rhythm and rate control groups were 4.5 years and seven, respectively. After the consultations, researchers concluded a significant decrease in hospitalizations in the rhythm control group. The incidence of heart failure-related hospitalizations among all patients with atrial fibrillation was 6% per year. Compared to the rate control group, the rhythm control group had a much-reduced yearly incidence of HF hospitalization (3% vs. 13%; p = 0.001) [83].

Challenges and future directions

Fortunately, surgical intervention and treatments have significantly improved the long-term survival of individuals with ToF. However, acquired conditions and late-term complications exist for adults with repaired ToF, such as pulmonary regurgitation, pulmonary stenosis, arrhythmia, and right ventricular dysfunction [84].

Restoring blood viscosity while conserving circulation in ToF patients with secondary polycythemia can be challenging. Isovolumetric venesections are beneficial in treating patients with symptomatic hyperviscosity but need to be monitored due to the increased risk of developing iron deficiency.

In addition to this, there is a lack of sufficient research regarding ToF in adult patients with secondary polycythemia. Case studies of secondary polycythemia are typically conducted on the patient population with hypoxia due to a condition other than ToF. Some of the cited conditions linked to secondary polycythemia include chronic obstructive pulmonary disease (COPD), chronic tobacco use, and hypoventilation syndromes. Further investigation is essential to improve therapeutic guidelines for patients with repaired ToF who develop secondary polycythemia.

Emerging research

The objective of a retrospective cohort study concluded that there was an increased risk of complications in adults with repaired ToF after receiving a PVR or TAP compared to other interventions. The researchers analyzed the outcomes of patients who underwent a valve-sparing (VS) procedure compared to patients who received a TAP. Specifically, these participants were categorized using a propensity score based on preoperative factors in a 1:1 ratio. The data collected was analyzed over 13 months. They concluded that the ToF patients who received valve-sparing (VS) surgery had a significantly increased 30-year survival rate compared to ToF patients who underwent TAP. Along with an increased survival rate, the same patients had fewer cardiovascular interventions and pulmonary valve replacements [85]. 

There are more outstanding beneficial outcomes in patient care when there is less hyperspecialization in medical practice. This emphasizes the importance of managing ToF with multiple disciplinary teams due to its highly variable presentation. Cardiology physicians are advised to consult hematology and interventional radiology specialists to assess the patient’s risk for secondary polycythemia and thrombus formation. Patients with heart failure frequently experience pulmonary and renal complications. Therefore, discussions should include physicians within the nephrology and pulmonology departments. Overall, the efficiency of decision-making and quality outcomes are enhanced when an interdisciplinary team manages a patient [86].

## Conclusions

Tetralogy of Fallot is a complex congenital heart disease characterized by four primary lesions: right ventricular outflow tract obstruction, ventricular septal defect, right ventricular hypertrophy, and overriding of the aorta. Advances in understanding developmental anomalies and surgical management have significantly improved mortality rates and life expectancy in patients with repaired TOF. However, the situation with patients with pulmonary regurgitation, valve dilatation of the aortic root, and TOF should be approached cautiously. Standard approaches like ECG and TTE should be implemented before and after the operation to complement the annual TTE adjustment. The base comprising the whole management work is non-pharmaceutical treatment such as weight reduction, stopping smoking, and exercising. Implementing dietary and lifestyle changes, such as reducing salt consumption, can benefit individuals experiencing volume overload. Physicians have carefully examined and considered the degree of right ventricular dysfunction, severity of pulmonary insufficiency, and residual outflow tract obstruction in patients with ToF. There is a fairly clear understanding of the pathogenesis between patients with ToF, heart failure, and secondary polycythemia. Despite the considerable amount of research conducted on adults with ToF, further investigation is required. We hope future studies are conducted to investigate the impact of biomarkers, such as IGFBP-7 and FABP4, on disease processes. There is a promising outlook that future research will address these topics and beneficially impact symptomatic adults with ToF.
